# Does diabetes modify the triglyceride–glucose index associated with cardiovascular events and mortality? A meta-analysis of 50 cohorts involving 7,239,790 participants

**DOI:** 10.1186/s12933-025-02585-z

**Published:** 2025-01-27

**Authors:** Jun Zhang, Qiye Zhan, Zhihao Deng, Ling Lin, Zhaolan Feng, Huabin He, Deju Zhang, Huilei Zhao, Xiang Gu, Xiaoping Yin, Peng Yu, Xiao Liu

**Affiliations:** 1 Department of Cardiology, Affiliated Hospital of Jiujiang University, Jiujiang, China; 2 Fujian Medical University, Fuzhou, Fujian China; 3 Department of Endocrinology and Metabolism, The Second Affiliated Hospital of Nanchang University, Nanchang, Jiangxi China; 4 Department of Cardiology, Jiujiang City Key Laboratory of Cell Therapy, JiuJiang NO.1 People’s Hospital, Jiujiang, Jiangxi China; 5 Food and Nutritional Sciences, School of Biological Sciences, The University of Hong Kong, Hong Kong, China; 6 Department of Anesthesiology, The Third Hospital of Nanchang, Nanchang, Jiangxi China; 7 Department of Neurology, Affiliated Hospital of Jiujiang University, Jiujiang, China; 8 Jiujiang Clinical Precision Medicine Research Center, Jiujiang, China; 9 Department of Cardiology, Sun Yat-sen Memorial Hospital of Sun Yat-sen University, Guangzhou, Guangdong China; 10 Cardiovascular & Metabolic Disorders Program, Duke-National University of Singapore Medical School, Singapore, Singapore

**Keywords:** Cardiovascular disease, Diabetes mellitus, Mortality, Meta-analysis, Triglyceride–glucose index

## Abstract

**Introduction:**

Previous studies highlighted the association between the triglyceride–glucose (TyG) index and cardiovascular events in patients with diabetes. However, whether diabetes affects TyG-cardiovascular diseases (CVD) is still unclear. This study aimed to evaluate the association between the TyG index and CVD risk, stratified by diabetes status, as well as the potential modifying effect of diabetic status.

**Methods/design:**

The PubMed, Cochrane Library, and Embase databases were searched for studies on the associations between the TyG index and cardiovascular events and mortality in patients with and without diabetes from inception to December 2, 2024. The random effects model was employed to pool the effect sizes.

**Results:**

A total of 50 cohort studies (7,239,790 participants) were included. The mean age of participants was 31.46 years (diabetes mellitus [DM]: 65.18; non-DM: 31.23), and 40.66% of participants were female (DM: 36.07%; non-DM: 40.70%). The associations between the TyG index and cardiovascular events (HR: 1.72 vs. 1.55, *P* = 0.55), major adverse cardiovascular and cerebrovascular events (HR: 2.02 vs. 1.91, *P* = 0.84), stroke (HR: 1.46 vs. 1.39, *P* = 0.77) and cardiovascular death (HR: 1.85 vs. 1.60, *P* = 0.56) were similar among DM and non-DM individuals. However, the associations between the TyG index and ischemic heart disease (IHD) (HR: 2.20 vs. 1.57, *P* = 0.03) as well as all-cause mortality (HR: 1.94 vs. 1.24, *P* = 0.01) were stronger in DM patients than in non-DM patients.

**Conclusion:**

TyG index showed association with cardiovascular events, mortality, and all-cause mortality independent of diabetic status, with low to moderate certainty. The associations for IHD and all-cause death were stronger in diabetic patients than in individuals without diabetes. Future studies should explore the role of diabetes in the TyG index-associated CVD outcomes and mortality.

**Graphical abstract:**

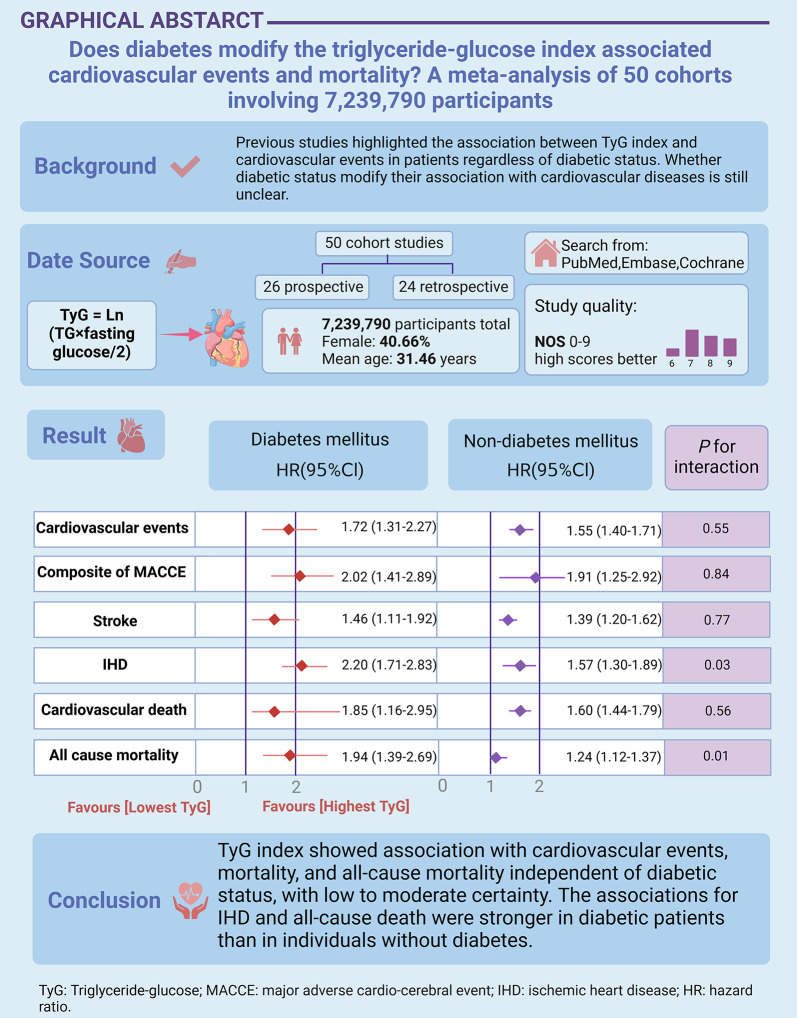

**Supplementary Information:**

The online version contains supplementary material available at 10.1186/s12933-025-02585-z.

## Introduction

Insulin resistance (IR) is acknowledged as a primary pathophysiological factor that drives the development of type 2 diabetes mellitus (T2DM) [1, 2]. The hyperinsulinemic–euglycemic (HIEG) clamp is considered the gold standard for assessing IR [3]. However, despite its status as the definitive method, the HIEG clamp test has several limitations because of its complexity and time-consuming nature. Several alternative indicators are employed to measure IR, such as the quantitative insulin sensitivity check index, the homeostasis model assessment-insulin resistance, and the triglyceride-to-high-density lipoprotein cholesterol ratio. Among these indices, the triglyceride-glucose (TyG) index, which is calculated from triglyceride and fasting blood glucose concentrations are readily accessible and cost-effective. Moreover, it demonstrates high sensitivity and specificity for assessing IR in comparison to the HIEG clamp test [4]. In addition to its role in IR evaluation, previous studies, including our own, have shown that the TyG index is associated with multiple diseases, including cardiovascular disease (CVD), metabolic syndrome, cancer, metabolic-associated fatty liver, kidney diseases, and osteoarthritis [5–13]. Moreover, increasing evidence suggests a significant association between the TyG index and the incidence of cardiovascular disease in both diabetic and nondiabetic individuals [14]. CVD ranks as one of the leading contributors to mortality in individuals diagnosed with diabetes. The TyG index has been extensively studied in diabetic populations and is recognized as a valuable tool for identifying adverse cardiovascular health outcomes. Research has shown that the TyG index correlates with the progression of atherosclerosis and is associated with major cardiovascular events, such as myocardial infarction and stroke, in patients with diabetes [15]. Within the nondiabetic population, multiple studies have shown that the TyG index is associated with cardiovascular events and mortality [16–46]. However, as a surrogate marker of IR, it remains unclear whether the association between the TyG index and cardiovascular disease (CVD) differs between individuals with and without diabetes. To date, no studies have systematically evaluated how diabetes affects the relationship between TyG and specific cardiovascular outcomes, leaving a critical gap in understanding its clinical implications in this high-risk group.

Given this background, this meta-analysis aimed to explore the associations between the TyG index and the risk of cardiovascular events and mortality in both diabetic and nondiabetic patients, as well as any potential differences.

## Methods

This protocol was registered with PROSPERO, the international prospective register of systematic reviews, with the registration number CRD42024506648. This meta-analysis reported the results following the Preferred Reporting Items for Systematic Reviews and Meta-Analyses (PRISMA) (Online Table S1) [47].

### Literature search

Two authors (X.L. and QY.Z.) independently conducted searches of PubMed, EMBASE and the Cochrane Library databases for articles published from inception to December 2, 2024, with no restrictions on language. The keyword search terms included the following: (1) For the TyG index: ‘TyG index’ OR ‘triglyceride glucose index’ OR ‘triglyceride-glucose index’ OR ‘triglyceride and glucose index’ OR ‘triacylglycerol glucose index’. (2) For CVD: ‘cardiovascular diseases’ OR ‘cardiovascular event’ OR ‘coronary artery disease’ OR ‘coronary disease’ OR ‘heart failure’ OR ‘stroke’ OR ‘myocardial infarction’ OR ‘mortality’ OR ‘death’. We also manually retrieved relevant reviews and meta-analyses. The comprehensive search strategy is available in Online Table S2.

### Study selection

Following the identification of potentially relevant articles via the search procedure described above, the studies were managed, and duplicates were eliminated. The initial screening involved assessing titles and abstracts, which was then followed by a review of the full texts. Any discrepancies were resolved through discussions until consensus was reached among the authors.

Studies were eligible for inclusion if they satisfied all of the following criteria: (1) adult (age > 18 years) with or without diabetes. (2) Studies reporting associations between TyG levels and cardiovascular events and mortality. Cardiovascular events were defined as cardiovascular, cerebrovascular, and/or cardiovascular death, including individual or combined occurrences of myocardial infarction, coronary artery disease (CAD), major cardiovascular events, transient ischemic attack, stroke, and ischemic heart events (Online Table S3). IHD was defined as individual or combined occurrence of myocardial infarction and CAD. (3) Cohort studies. (4) Follow-up period ≥ 1 year. In addition, articles meeting the following criteria were excluded: systematic reviews, case–control studies, meta-analyses, reports, or comments. Studies reporting on patients in the intensive care unit were excluded. For duplicate studies, we selected the one with the most recent publication date, the greatest number of participants, the highest methodological quality, and the most comprehensive data. All six studies were sourced from Beijing Anzhen Hospital because they had different outcomes, types of populations, or statistical methods (categorical or continuous variables) [21, 29, 46, 48–50].

### Data collection and quality assessment

Two researchers (QY.Z. and X.L.) independently extracted the data from the eligible studies and performed the quality assessment. Any differences were addressed through discussion or consultation with a third author (X.L.).

The extracted data encompassed research characteristics (including author name, publication year, country, study design, and follow-up duration), patient demographics (including sample size, sex, age, and population source), outcome measures and results, and any adjustments made.

The quality of the eligible studies was assessed utilizing the Newcastle–Ottawa Scale (NOS) [51].

### Statistical analysis

Pooled adjusted hazard ratios (HRs) with a random effects model, along with their corresponding 95% confidence intervals (CIs), were used to estimate the effect sizes. If the outcome indicator of the included study was the odds ratio (OR), it was analyzed as an HR when the incidence of outcomes was low (< 10%) [52].

The TyG index was calculated using the formula ln (fasting triglycerides (mg/dl) × fasting blood glucose (mg/dl)/2). For categorical variables, the highest TyG index was pooled with the lowest TyG index. A supplementary analysis was conducted to compare the median TyG index level group (third highest if four groups were present) with the lowest TyG index levels. For continuous variables (per 1-unit increment), HRs were extracted for analysis. Tau² (τ2) and the p value of the Q test were utilized to assess the degree of heterogeneity [53]. Significant heterogeneity was identified when the p-value of the Q test was low (< 0.10). I² served as an indicator of inconsistency among the results. The quality and strength of evidence for each outcome were evaluated according to the Grading Recommendations Assessment, Development, and Evaluation (GRADE) method [54].

Subgroup analyses were conducted to evaluate the impact of mean age, glycemic control (effective vs. poor), sample size, follow-up duration, and study design on the associations between the TyG index and cardiovascular events. Sensitivity analyses were performed to examine the robustness of the pooled estimates by excluding each study one at a time. Publication bias was evaluated through Egger’s test and funnel plots when a specific outcome included more than ten studies [55]. If Egger’s test indicated publication bias, a trim-and-fill analysis was employed to evaluate the stability of the combined results. An effect was deemed significant when *P* < 0.05. Statistical analysis was conducted using RevMan software (version 5.4, The Cochrane Collaboration, Nordic Cochrane Center Copenhagen, Denmark) and R software (version 4.1.2, www.r-project.org).

## Results

### Literature search

**Fig. 1:**
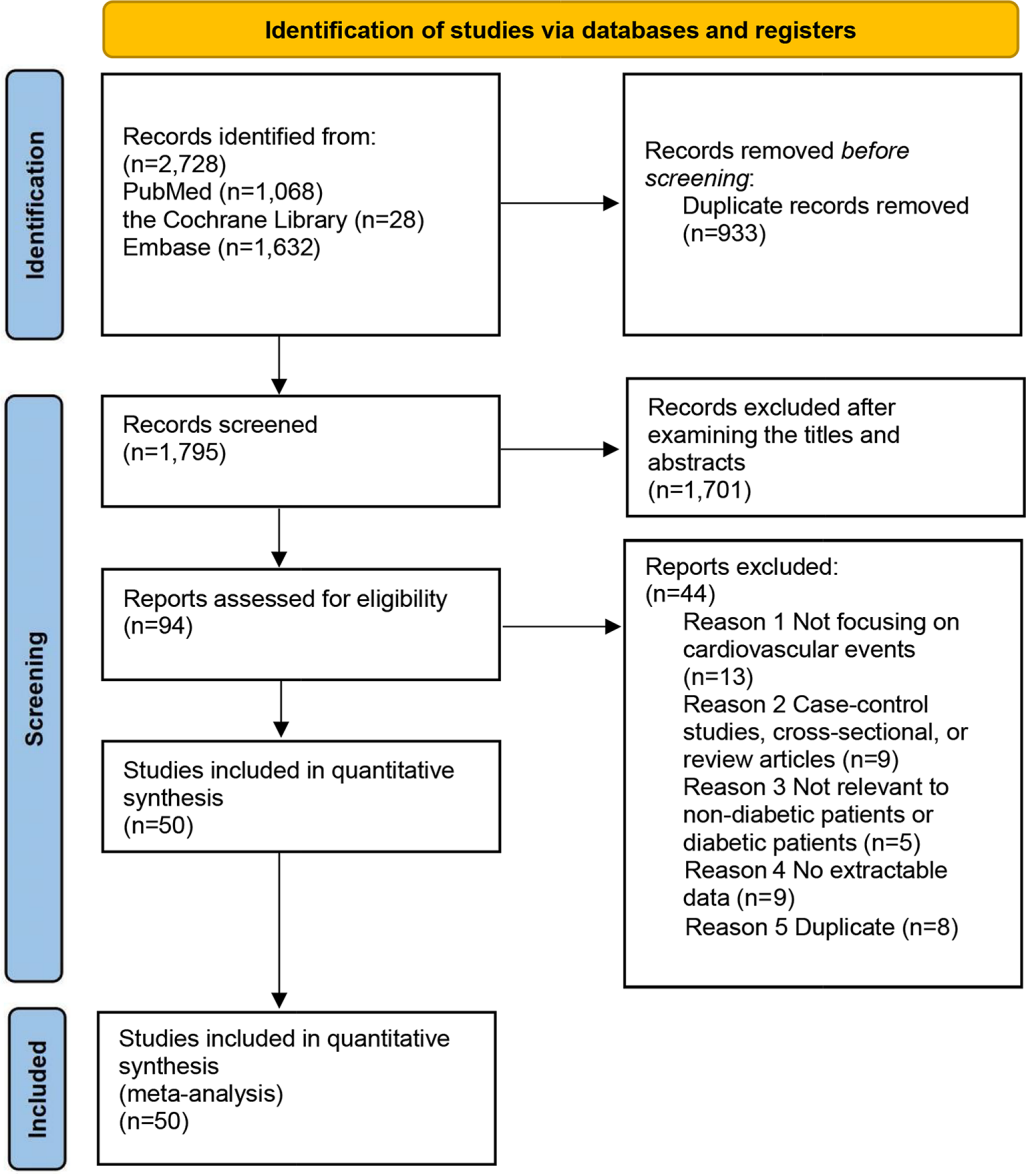
Flow chart of the study selection process in the meta-analysis of TyG index and risk of cardiovascular events and mortality in patients with or without diabetes

Figure 1 depicts the procedure followed during the database search. The initial search yielded 2,728 literature citations (PubMed = 1,068; Cochrane Library = 28; Embase = 1,632), which were reduced to 1,795 after removing duplicates and irrelevant studies. From these, 1,701 articles were excluded because of irrelevance after screening their titles and abstracts. Subsequently, 94 papers were subjected to full-text review, resulting in the exclusion of 44 articles for the following reasons: (a) cross-sectional, case–control studies, or review articles (*n* = 9); (b) not focused on cardiovascular events (*n* = 13); (c) not specifically focused on patients with or without diabetes (*n* = 5); (d) no extractable data (*n* = 9); and (e) duplicated studies (*n* = 8). Ultimately, 50 cohort studies were included in the meta-analysis [6, 14, 16–46, 48–50, 56–69]. The excluded studies (*n* = 44) are shown in Online Table S4.

### Study characteristics and quality evaluation

Table 1 presents an overview of the characteristics of the included studies. Thirty-two studies included participants with diabetes [6, 14, 25, 32–38, 40, 43–46, 48–50, 56–69], whereas 31 studies involved individuals without diabetes [16–46]. The participants had a mean age of 31.46 years (diabetes mellitus [DM]: 65.18; non-DM: 31.23), and 40.66% of participants were female (DM: 36.07%; non-DM: 40.70%). Among these studies, 26 were prospective cohort studies [16–20, 22–27, 31, 32, 35, 36, 40, 41, 43, 45, 56, 59, 62, 63, 65, 67, 69], and the others were retrospective [6, 14, 21, 28–30, 33, 34, 37–39, 42, 44, 46, 48–50, 57, 58, 60, 61, 64, 66, 68]. Among the 50 eligible studies published between 2019 and 2024, 43 involved cardiovascular events, 22 involved major adverse cardiovascular and cerebrovascular events (MACCEs), 14 involved stroke, 13 involved ischemic heart disease (IHD), 6 involved cardiovascular death, and 25 involved all-cause mortality. Twenty-five studies had follow-up periods of 5 years [6, 16, 18, 19, 25–28, 30, 32–38, 40, 41, 43, 56, 59, 62, 67, 68]. Most studies adjusted for age and sex, whereas adjustments for additional confounding variables varied significantly.

**Table 1:** Characteristics of included studies in this meta-analysis of triglyceride-glucose index and risk of cardiovascular events and mortality in patients with or without diabetes

Authors, year, country	Study design, follow up	Source of participant/sample	Mean age (years), Female (%)	Endpoint	Diagnostic criteria for endpoint	TyG index	HR/OR (95%CI)	Adjustments
Abuduaini et al. [60]	Retrospective cohort/4.62 years	First Affiliated Hospital of Xinjiang Medical University (patients with DM and ischemic cardiomyopathy)/1514	65.26, 26.10	MACCE (chest pain, acute MI, HF, cardiogenic shock, malignant arrhythmias, cerebral infarction, gastrointestinal bleeding, all-cause death)	ICD	≤ 7.21 7.21–7.89 ≥ 7.89	Ref HR: 2.15 (1.68–2.75) 4.86 (3.84–6.14)	Age, BMI, neutrophil, lymphocyte, PLT, UA, HDL-C, LDL-C, ALP, LVEF, gender, TC, HT his, ethnicity, medication.
				AMI		≤ 7.21 7.21–7.89 ≥ 7.89	Ref HR: 4.24 (1.37–13.18) 4.44 (1.42–13.87)	
				HF		≤ 7.21 7.21–7.89 ≥ 7.89	Ref HR: 2.66 (1.16–6.07) 7.33 (3.42–15.71)	
				All-cause death		≤ 7.21 7.21–7.89 ≥ 7.89	Ref HR: 2.02 (1.54–2.65) 4.50 (3.48–5.83)	
Chen et al. [48]	Retrospective cohort/2 years	Beijing Anzhen Hospital (patients with T2DM who underwent isolated OPCABG surgery between September 2017 to June 2019)/1578	62.90, 27.30	All-cause death	ICD	< 8.85 8.85–9.38 ≥ 9.38	Ref HR: 0.95 (0.27–3.27) 0.61 (0.15–2.43)	Age, sex, BMI, current smoking, hypertension, previous MI, previous stroke, past PCI, cardiac failure, CKD, preoperative LVEF, insulin dependence, LDL-c, HDL-C, diagnosis, extent of CAD, left main disease, complete revascularization, and use of IABP
				MACCE (all-cause death, nonfatal MI, nonfatal stroke and symptomdatic graft failure)		< 8.85 8.85–9.38 ≥ 9.38	Ref HR: 1.73 (1.09–2.73) 2.13 (1.35–3.38)	
				Non-fatal stroke		< 8.85 8.85–9.38 ≥ 9.38	Ref HR: 1.41 (0.54–3.69) 2.08 (0.81–5.33)	
				Non-fatal MI		< 8.85 8.85–9.38 ≥ 9.38	Ref HR: 3.47 (1.14–10.56) 4.35 (1.44–13.18)	
Liu et al. [63]	Prospective cohort/1 year	China National Stroke Registry II (acute IS patients with type 2 diabetes)/3359	65.00, 40.80	Ischemic stroke recurrence	ICD	0.40–1.60 1.60-2.00 2.00-2.50 2.50–3.30	Ref HR: 1.01 (0.72–1.43) 1.41 (0.97–2.03) 1.06 (0.60–1.85)	Age, sex, BMI, SBP, DBP, NIHSS on admission, HbA1c, TC, TG, HDL-C, LDL-C, FBG, intravenous thrombolysis, medical history of ischemic stroke, ICH, AF, medication history of Statins, antidiabetics, antihypertension, medication at discharge of antidiabetic agents, statins, antihypertensive agents and TOAST subtypes
				All-cause death		0.40–1.60 1.60-2.00 2.00-2.50 2.50–3.30	Ref HR: 1.14 (0.76–1.70) 1.70 (1.06–2.74) 1.67 (0.76–3.69)	
Shen et al. [62]	Prospective cohort/10 years	Department of Cardiology in Chinese PLA General Hospital (ACS patients ≥ 80 years old with type 2 diabetes)/231	81.58, 32.47	All-cause mortality	ICD	Per SD (0.67) 7.98–8.62 8.62–9.15 9.15–10.18	HR: 1.44 (1.13–1.83) Ref HR: 1.62 (0.87–3.02) 2.04 (1.09–3.81)	Age, sex, BMI, SBP, DBP, LVEF, Gensini score, hypertension, hyperlipidemia, previous MI, previous stroke, CKD, current smoking, TC, LDL-C, HDL-C, eGFR, UA, aspirin, clopidogrel, statin, β-blocker, ACEI/ARB, LM lesion, multivessel lesion and treatment
Su et al. [6]	Retrospective cohort/5.93 years	Kaohsiung Medical University Hospital (patients with T2DM)/3524	61.68, 50.90	Cardiovascular events (CAD, unstable angina, MI, stroke, peripheral arterial disease, and CV-related death)	ICD-9	Per 1 unit	HR: 1.52 (1.14–2.03)	Hemoglobin A1c and significant variables in the univariable analysis except fasting glucose
Wang et al. [45]	Retrospective cohort/3 years	Tianjin Chest Hospital (patients with T2DM and ACS)/2531	66.30, 44.10	All-cause death	ICD	≤ 8.85 8.85–9.38 ≥ 9.38	Ref HR: 1.07 (0.69–1.65) 1.27 (0.83–1.94)	Age, male, smoker, previous MI, previous CABG, BMI, AMI, LVEF, left main disease, multi-vessel disease, HbA1c, hs-CRP, statin, insulin
				Non-fatal MI		≤ 8.85 8.85–9.38 ≥ 9.38	Ref HR: 1.59 (0.94–2.70) 1.71 (1.01–2.90)	
				Non-fatal stroke		≤ 8.85 8.85–9.38 ≥ 9.38	Ref HR: 1.24 (0.55–2.78) 2.07 (0.98–4.34)	
				MACE (all-cause death, non-fatal MI and non-fatal stroke)		Per 1 unit ≤ 8.85 8.85–9.38 ≥ 9.38	1.45 (1.21–1.75) Ref HR: 1.27 (0.93–1.72) 1.54 (1.14–2.08))	
Wang et al. [65]	Prospective cohort/NA	Cardiovascular Center Beijing Friendship Hospital Database Bank (patients with DM who were diagnosed with ACS)/5046	65.60, 38.00	MACCE (all-cause death, non-fatal MI, non-fatal stroke)	ICD	Per 1 unit < 8.75 8.75–9.34 > 9.34	HR: 1.18 (1.05–1.32) Ref HR: 1.15 (0.97–1.38) 1.28 (1.06–1.54)	NT-pro, BNP, age, sex, BMI, diagnosis of AMI, history of hypertension, history of dyslipidemia, history of MI, history of arrhythmia, SBP, LVEF, eGFR, hs-CRP, LDL-C, smoking status, and in-hospital treatments
Xiong et al. [61]	Retrospective cohort/1.57 years	Third People’s Hospital of Chengdu (patients with T2DM diagnosed with CHD and undergoing PCI)/633	68.02, 32.80	MACE (all-cause death, nonfatal MI, and unplanned repeat revascularization)	ICD	Per 1 unit	HR: 1.75 (1.22–2.50)	Age, BMI, ACS, heart rate, BNP, Scr, bSS, rSS, LVEF, diuretics, and insulin
Zhao et al. [49]	Retrospective cohort/3 years	Beijing Anzhen Hospital (patients with T2DM who were diagnosed with NSTE-ACS and treated with elective PCI between January and December 2015)/798	60.90, 31.70	All-cause death	ICD	Per 1 unit < 9.18 ≥ 9.18	HR: 0.43 (0.11–1.66) Ref HR: 0.87 (0.18–4.26)	Age, sex, BMI, SBP, DBP, smoking, drinking, duration of diabetes, dyslipidemia, prior MI, PCI, stroke, PVD, diagnosis, TC, HDL-C, eGFR, HbA1c, LVEF, SYNTAX score, LM treatment, DCB use, complete revascularization, number of stents, DAPT, DAPT interruption in 12 months, statins, statins interruption in 12 months, oral hypoglycemic agents and insulin
				Non-fatal MI		Per 1 unit < 9.18 ≥ 9.18	HR: 3.33 (1.73–6.42) Ref HR: 2.27 (0.89–5.72)	
				MACE (all-cause death, non-fatal MI, and ischemia-driven revascularization)		Per 1 unit < 9.18 ≥ 9.18	HR: 3.21 (2.40–4.29) Ref HR: 4.06 (2.73–6.04)	
Yao et al. [62]	Retrospective cohort/3.42 years	Affiliated Hospital of Jiangsu University (patients with T2DM)/858	67.13, 44.00	All-cause mortality	ICD	Per 1 unit ≤ 8.70 8.70–9.20 9.20–9.67 ≥ 9.67	HR: 1.89 (1.29–2.76) Ref HR: 0.92 (0.37–2.29) 1.55 (0.66–3.65) 3.36 (1.37–8.28)	Age, duration of diabetes, hypertension, aortic calcification, ACEI/ARB medication history, albumin, TG, and TC.
Si et al. [57]	Retrospective cohort/NA	Department of Cardiology, Affiliated Hospital of Chengde Medical College (patients with CAD with T2DM)/680	60.00, 40.30	CAD	ICD	< 8.0 ≥ 8.0	Ref OR: 2.64 (1.15–6.05)	BMI, smoking, hypertension, dyslipidemia, ischemic stroke, pulse pressure
Colladant et al. [25]	Prospective cohort/9.1 years	Transplant Unit of the CHU of Besancon (kidney transplant recipients with DM)/236	NA	Cardiovascular events (MI, coronary revascularization, stroke)	ICD	per 0.1	HR: 1.23 (1.01–1.64)	Age, eGFR, sex
		(kidney transplant recipients without DM)/479	NA	Cardiovascular events (MI, coronary revascularization, stroke)		Per 0.1	HR: 1.93 (1.19–3.56)	
Dong et al. [46]	Retrospective cohort/3.5 years	Beijing Anzhen Hospital (patients with a history of CABG and DM who had PCI between January 2010 and September 2020)/533	NA	MACCE (all-cause death, nonfatal stroke, nonfatal MI, and unplanned repeat revascularization)	ICD	Per 1 unit	HR: 1.47 (1.00-2.16)	Age, male, BMI, SBP, current smoking, family history of CAD, hypertension, dyslipidemia, T2DM, prior MI, prior PCI, prior stroke, PAD, HF, CKD, clinical diagnosis, Hb, hs-CRP, eGFR, TC, LDL-C, HDL-C, HbA1c, LVEF, DAPT, ACEI/ARB, ARNI, antidiabetic agents, statins at discharge, LM disease, multivessel disease, CTO, thrombotic disease, in-stent restenosis, target vessel selection, PTCA, number of stents, interval time from CABG to PCI, PCI success
		Beijing Anzhen Hospital (patients with a history of CABG and without DM who had PCI between January 2010 and September 2020)/625	NA	MACCE (all-cause death, nonfatal stroke, nonfatal MI, and unplanned repeat revascularization)		Per 1 unit	HR: 1.19 (0.80–1.79)	
Wei et al. [67]	Prospective/6.92 years	Foot Disease Center of the Guangxi Academy of Medical Sciences and Guangxi Zhuang Autonomous Region People’s Hospital (patients with diabetic foot ulcers)/960	63.23, 32.50	MACCE (all-cause mortality, non-fatal MI, coronary artery revascularization and non-fatal stroke)	ICD	< 9.12 ≥ 9.12	Ref HR: 1.81 (1.40–2.33)	Age, sex, hypertension, previous CHD, previous CVD, PAD, DPN, DN, smoking, drinking, duration of diabetes, SBP, and TC
				Non-fatal MI		< 9.12 ≥ 9.12	Ref HR: 2.19 (1.16–4.10)	
				Non-fatal stroke		< 9.12 ≥ 9.12	Ref HR: 1.76 (1.22–2.55)	
Huang et al. [68]	Retrospective cohort/5.25 years	Foot Healthcare Centre of Guangxi People’s Hospital (patients with diabetic foot ulcers)/555	64.49, 31.70	All-cause mortality	ICD	Per 1 unit ≤ 8.75 8.76–9.33 ≥ 9.34	HR: 1.73 (1.34–2.24) Ref HR: 1.69 (1.01–2.81) 2.77 (1.68–4.57)	Gender, age, hypertension, CHD, CVD, duration of diabetes, SBP, DBP, Hb, Cr
Lin et al. [50]	Retrospective cohort/1.83 years	Beijing Anzhen hospital (patients with T2DM and CTO underwent PCI between January 2018 to December 2019)/681	59.16, 17.30	MACCE (overall death, nonfatal MI, and unplanned revascularization)	ICD	Per 1 unit	HR: 1.70 (1.25–2.30)	Age, BMI, SBP, previous MI, previous PCI, TC, LDL-C, TG, FBG, HbA1c, eGFR, uric acid, and insulin
Park et al. [32]	Prospective cohort/13.7 years	Ansung-Ansan cohort database (participants with DM)/918	NA	Cardiovascular disease (MI, angina pectoris, HF, peripheral artery disease, and/or stroke)	ICD	< 8.38 8.38–8.80 > 8.80	Ref HR: 0.77 (0.44–1.33) 1.01 (0.56–1.82)	Age, sex, BMI, physical activity, smoking status, current drinker, CRP, FPG, hypertension, and dyslipidemia
		(participants without DM)/6890	NA	Cardiovascular disease (MI, angina pectoris, HF, peripheral artery disease, and/or stroke)		< 8.38 8.38–8.80 > 8.80	Ref HR: 1.28 (0.95–1.71) 1.42 (1.06–1.90)	
Qin et al. [58]	Retrospective cohort/1.92 years	First Affiliated Hospital of Zhengzhou University (ACS patients with T2DM who underwent PCI)/899	59.98, 35.20	MACE (all-cause death, malignant arrhythmia, non-fatal MI, target vessel reconstruction, angina pectoris requiring hospitalization, and acute HF)	ICD	Per 1 unit	HR: 1.81 (1.48–2.20)	LVEF, FBG, glycated hemoglobin, urea and uric acid, TC, LDL-C, HDL-C and TG levels, NT-pro BNP, fibrinogen, complete blood count, and WBC count
Tai et al. [59]	Prospective cohort/8.8 years	ACCORD/ACCORDION trial (patients with T2DM)/10,196	62.77, 38.52	MACE (non-fatal MI, non-fatal stroke, and death from CV causes)	ICD	Per 1 unit 6.87-9.00 9.01–9.47 9.47–9.95 9.95–13.36	HR: 1.19 (1.11–1.28) Ref HR: 1.05 (0.91–1.21) 1.08 (0.99–1.18) 1.13 (1.06–1.20)	Age, sex, previous cardiovascular event, race, BMI, education, SBP, diastolic blood pressure, eGFR, HbA1c, total plasma cholesterol, plasma LDL-C, live alone, duration of diabetes, depression, statins, biguanide, aspirin, ACEI/ARB, and insulin
				Major coronary events (CV death, non-fatal MI, or unstable angina)		Per 1 unit 6.87-9.00 9.01–9.47 9.47–9.95 9.95–13.36	HR: 1.22 (1.14–1.31) Ref HR: 1.22 (1.06–1.41) 1.12 (1.03–1.22) 1.17 (1.10–1.24)	
Zhang et al. [14]	Retrospective cohort/2.23 years	Cardiovascular Center of Beijing Friendship Hospital Database (patients with T2DM and AMI)/1932	65.40, 31.50	MACCE (all-cause death, non-fatal MI, non-fatal stroke, cardiac rehospitalization, and revascularization)	ICD	≤ 8.91 8.91–9.54 ≥ 9.54	Ref HR: 1.58(1.33–1.88) 2.32 (1.92–2.80)	Age, BMI, history of stroke and PCI, antiplatelet agent used before admission, WBC, hemoglobin, albumin, eGFR, LVEF, angiography findings, in-hospital treatment and hypoglycemic agents
				All cause death		≤ 8.91 8.91–9.54 ≥ 9.54	Ref HR: 1.67 (1.24–2.25) 2.35 (1.72–3.20)	
				CV death		≤ 8.91 8.91–9.54 ≥ 9.54	Ref HR: 1.60 (1.11–2.30) 2.71 (1.92–3.83)	
				Non-fatal MI		≤ 8.91 8.91–9.54 ≥ 9.54	Ref HR: 1.37 (0.90–2.10) 2.02 (1.32–3.11)	
				Non-fatal stroke		≤ 8.91 8.91–9.54 ≥ 9.54	Ref HR: 0.94 (0.50–1.78) 1.39 (0.73–2.63)	
Zhao et al. [56]	Prospective cohort/6.83 years	National Health and Nutrition Examination Survey (patients with T2DM)/2998	64.00, 46.83	All cause death	ICD-10	≤ 8.72 8.72–9.15 9.15–9.65 > 9.65	Ref HR: 0.80 (0.65–0.98) 0.88 (0.72–1.08) 1.14 (0.91–1.41)	Age, sex, education level, PIR, BM, race/ethnicity, smoking status, alcohol consumption status, CHD, HF, heart attack, stroke, angina, hypertension, hyperlipidemia, glucose-lowering medication use, HbA1c, LDL-C and TC
				Cardiovascular mortality (rheumatic heart disease, hypertensive heart disease, ischemic heart disease, acute MI, pericardial disease, and acute myocarditis and HF)		≤ 8.72 8.72–9.15 9.15–9.65 > 9.65	Ref HR: 0.69 (0.48-1.00) 0.72 (0.50–1.04) 1.04 (0.70–1.53)	
Lin et al. [69]	Prospective cohort/3.0 years	Fuwai Hospital from January 2017 to December 2018 (diabetic patients with angiograph-proven CAD)/9996	60.29, 24.90	Cardiovascular events (cardiovascular death, nonfatal MI and nonfatal stroke)	ICD	Per 1 unit < 8.90 8.90–9.40 ≥ 9.40	HR: 1.78 (1.35–2.35) Ref HR: 1.10 (0.84–1.45) 1.40 (1.02–1.94)	Age, male sex, BMI, ACS presentation, family history of CAD, previous MI, previous revascularization, hypertension, previous stroke, PAD, current smoker, LVEF, serum creatinine, TC, HDL-C, LDL-C, hsCRP, SYNTAX score, CTO lesion, aspirin use, statins use and insulin use
				MACEs (cardiovascular death and nonfatal MI)		Per 1 unit < 8.90 8.90–9.40 ≥ 9.40	HR: 1.93 (1.43–2.60) Ref HR: 1.15 (0.85–1.54) 1.55 (1.09–2.20)	
Li et al. [36]	Prospective cohort/10.2 years	National Health and Nutrition Examination Survey between 1999 and 2018 (patients with DM)/5558	NA	All-cause mortality	ICD-10	Per 1 unit	HR: 1.26 (1.07–1.48)	Age, sex, ethnicity, SBP, BMI, smoking status, alcohol consumption, ASCVD, COPD, CHF, renal disease, cancer, TC, HDL-C, LDL-C, albumin, eGFR, statin use, and insulin or antihy perglycemic agents
				Cardiovascular mortality		Per 1 unit	HR: 1.30 (1.01–1.65)	
		(patients without DM)/22,084	NA	All-cause mortality		Per 1 unit	HR: 1.28 (1.14–1.44)	
				Cardiovascular mortality		Per 1 unit	HR: 1.31 (1.05–1.57)	
Jia et al. [35]	Prospective cohort/2.96 years	Hebei General Hospital (patients with DM)/424	NA	All-cause mortality	ICD	Per 1 unit	HR: 1.21 (1.01–1.44)	Age, BMI, sex, DBP, smoking, IHD, AF, New York Heart Association classification, CKD, valvular heart disease, haemoglobin, urea, serum creatinine, NT-proBNP, TC, LDL-C, triglyceride, LVEF, angiotensin receptor inhibitor, ACEI, angiotensin receptor neprilysin inhibitor
		(patients without DM)/850	NA	All-cause mortality		Per 1 unit	HR: 0.96 (0.79–1.15)	
Zhao et al. [37]	Retrospective cohort/2.58 years	Dongfang Hospital of Beijing University of Chinese Medicine (patients with CHD and comorbid depression and T2DM)/292	NA	MACCE (all-cause death, stroke, MI, emergent coronary revascularization)	ICD	Per 1 unit	HR: 1.89 (1.34–2.66)	Age, sex, BMI, smoking, SBP, DBP, hypertension, T2DM, dyslipidemia, prior CVDs, prior PCI, prior stroke, family history of CVDs, TC, HDL-C, LDL-C, HbA1c, antiplatelet medication, ACEI/ARB, CCB, b-Blocker, antidiabetic agents, statins, antidepressants, benzodiazepines
		(patients without T2DM)/304	NA	MACCE (all-cause death, stroke, MI, emergent coronary revascularization)		Per 1 unit	HR: 3.52 (2.10–5.90)	
Cai et al. [33]	Retrospective cohort/3.4 years	Health centers and community service centers in nine cities in Fujian Province (patients with DM at high risk of CVD)/7966	NA	All-cause mortality	ICD	Per 1 unit	HR: 1.99 (1.41–2.79)	Age, gender, hypertension, dyslipidemia, current smoking, HDL-C, LDL-C, BMI, and SBP
		(patients without DM at high risk of CVD)/27,489	NA	All-cause mortality		Per 1 unit	HR: 1.26 (0.59–2.67)	
Huang et al. [34]	Retrospective cohor/3.17 years	REal-world Data of CARdiometabolic ProtEcTion study (patients diagnosed with moderate to severe AS and DM)/64	NA	All-cause mortality	ICD	Per 1 unit	HR: 2.63 (0.95–7.76)	Sex, age, BMI, L-DLC, SBP, DBP, smoking status, drinking status, aortic stenosis severity, pulmonary arterial hypertension, LV ejection fraction, bicuspid aortic valve, aortic valve replacement, CHD, antiplatelets, and statins use
		(patients diagnosed with moderate to severe AS without DM)/253	NA	All-cause mortality		Per 1 unit	HR: 1.51 (0.91–2.50)	
Zhou et al. [38]	Retrospective cohor/3.9 years	PLA General Hospital (patients with DM and chronic HF)/2987	NA	All-cause mortality	ICD-10	< 8.40 8.40–8.93 ≥ 8.93	Ref HR: 1.45 (1.25–1.68) 1.95 (1.67–2.29)	Age, gender, BMI, smoking status, drinking status, hemoglobin, ALT, AST, TBil, albumin, eGFR, TC, LDL-C, HDL-C, cTnT, sodium, NT-proBNP, LVEF, NYHA classifcation, hypertension, AF, previous MI, angina, stroke, COPD, previous heart surgery, antiplatelet agents, lipid-lowering drugs, ACEI/ARB, ARNI, β-blocker, mineralocorticoid antagonists, diuretics, digoxin and hypoglycemic therapy
				Cardiovascular death		< 8.40 8.40–8.93 ≥ 8.93	Ref HR: 1.51 (1.24–1.83) 2.13 (1.74–2.60)	
		(patients with chronic HF without DM)/3710	NA	All-cause mortality		< 8.40 8.40–8.93 ≥ 8.93	Ref HR: 1.23 (1.04–1.45) 1.54 (1.27–1.86)	
				Cardiovascular death		< 8.40 8.40–8.93 ≥ 8.93	Ref HR: 1.43 (1.15–1.79) 1.66 (1.29–2.13)	
Hou et al. [40]	Prospective cohort/10.8 years	Kailuan Study (patients with DM)/4722	NA	All-cause mortality	ICD-10	Per 1 unit	HR: 1.16 (0.47–2.87)	Age, sex, educational background, smoking status, drinking status, snoring, SBP, history of hypertension, family history of CVD, use of antidiabetic agents, lipid-lowering agents, antihypertensive medications, TC, HDL-C, LDL-C, hs-CRP
				ASCVD (MI, ischemic stroke and revascularization therapy)		Per 1 unit	HR: 3.13 (1.01–9.83)	
		(patients without DM)/59,767	NA	All-cause mortality		Per 1 unit	HR: 1.32 (1.10–1.58)	
				ASCVD (MI, ischemic stroke and revascularization therapy)		Per 1 unit	HR: 1.45 (1.24–1.71)	
Fu et al. [39]	Retrospective cohort/2.7 years	Fuwai Hospital, between January 2017 and May 2022 (patients diagnosed with CAD and psoriasis that received coronary angiography without T2DM)/188	NA	MACEs (cardiac death, ACS, stroke, urgent revascularization, and HF)	ICD	< 8.40 ≥ 8.40	Ref HR: 2.07 (1.08–3.97)	Age, sex, smoking status, hypertension, stroke, and admission for ACS, use of RAASI, platelet count, hsCRP levels, LDL-C, LVEF, presence of LM/TVD, angulated lesion, and PCI therapy
Qiu et al. [44]	Retrospective cohort/2.9 years	Fujian Provincial Hospital (women with AMI and DM)/93	NA/100	MACCEs (all cause mortality, MI, repeat revascularization, rehospitalization for HF, and stroke)	ICD	Per 1 unit	HR: 2.07 (1.08–3.97)	Age, sex, smoking status, SBP, and TC
		(women with AMI without DM)/227	NA/100	MACCEs (all cause mortality, MI, repeat revascularization, rehospitalization for HF, and stroke)		Per 1 unit	HR: 4.35 (2.65–7.12)	
(1)Lin et al. [43]	Prospective cohort/25.0 years	Third National Health and Nutrition Examination Survey between 1988 and 1994 (patients with DM)/1320	NA	All-cause mortality	ICD-10	Per 1 unit	HR: 1.22 (1.06–1.40)	Age, sex, BMI, race/ethnicity, educational level, poverty income ratio, smoking, alcohol use, physical activity, hypertension, cardiovascular disease history, and cancer history, insulin usage, antidiabetic drugs, and lipid-lowering drugs
		(patients without DM)/12,589	NA	All-cause mortality		Per 1 unit	HR: 1.06 (0.95–1.18)	
Xu et al. [45]	Prospective cohort/3.0 years	China Health and Retirement Longitudinal Study (patients with DM)/1583	NA	Stroke	ICD	< 8.25 8.25–8.61 8.62–9.06 ≥ 9.07	Ref OR: 1.01 (0.39–2.82) 0.91 (0.38–2.43) 0.77 (0.34–1.98)	Age grade, gender, marital, region, sleep, nap, smoke, drink, depression, hyperuricemia, blood urea nitrogen, LDL, TC, heart disease, hepatic disease, renal disease, digestive disease, arthritis, HP, and BMI
		China Health and Retirement Longitudinal Study (patients without DM)/9613	NA	Stroke		< 8.25 8.25–8.61 8.62–9.06 ≥ 9.07	Ref OR: 1.17 (0.86–1.58) 1.39 (1.03–1.88) 1.57 (1.14–2.17)	
Khalaji et al. [42]	Retrospective cohort/1.04 years	Tehran Heart Center (patients with ACS undergoing PCI and without prediabetes or diabetes)/4338	NA	MACCE (all-cause mortality, MI, stroke, target vessel revascularization, target lesion revas cularization, and CABG)	NA	Per 1 unit ≤ 8.54 8.55–8.93 8.94–9.39 > 9.39	HR: 1.06 (0.82–1.38) Ref HR: 1.03 (0.75–1.42) 0.92 (0.65–1.30) 1.06 (0.75–1.52)	Age, sex, LVEF, hypertension, BMI, waist circumference, LDL-C, HDL-C, creatinine, hemoglobin, family history of CAD, cigarette smoking, opium, type of ACS (STEMI, NSTEMI, or UA), and past medical histories of congestive HF, valvular heart disease, cerebrovascular disease, CPR, previous CABG, previous PCI, atrial fibrillation, STEMI, NSTEMI, UA, and SA
Blicher et al. [41]	Prospective cohort/15.4 years	MONItoring of trends and determinants in CArdiovascular disease project (patients without diabetes)/1970	NA, 50.2	MACE (stroke, IHD, HF, revascularization for PAD, and death from presumed cardiovascular causes)	NA	Per 1 unit	HR: 1.16 (1.03–1.31)	Age, sex, smoking status, SBP, and TC
Cho et al. [16]	Prospective cohort/7.4 years	National Health Information Database (community population without DM or GCVD)/6,675,424	30.68, 40.37	Stroke	ICD-10	< 8.13 8.13–8.54 8.54–8.98 ≥ 8.98	Ref HR: 1.03 (0.96–1.11) 1.12 (1.04–1.20) 1.25 (1.17–1.35)	Age, sex, BMI, smoking, alcohol consumption, physical activities, income, presence of hypertension, and TC concentration
				MI		< 8.13 8.13–8.54 8.54–8.98 ≥ 8.98	Ref HR: 0.98 (0.92–1.04) 1.01 (0.96–1.07) 1.26 (1.19–1.33)	
				All-cause death		< 8.13 8.13–8.54 8.54–8.98 ≥ 8.98	Ref HR: 0.99 (0.94-1.00) 1.01 (0.97–1.05) 1.15 (1.10–1.20)	
Hou et al. [17]	Prospective cohort study/1.0 year	China National Stroke Registry II (ischemic stroke patients without DM)/12,964	64.83, 34.83	All-cause death	ICD	< 8.33 8.34–8.73 8.74–9.20 > 9.21	Ref HR: 1.06 (0.90–1.25) 1.04 (0.87–1.24) 1.07 (0.88–1.30)	Sex, age, NIHSS score at admission, IV thrombolytic administration, prior/current smoking history, medical history, medications, and laboratory examination
				Stroke recurrence		< 8.33 8.34–8.73 8.74–9.20 > 9.21	Ref HR: 0.97 (0.80–1.18) 0.97 (0.79–1.19) 1.18 (0.96–1.45)	
Zhou et al. [28]	Retrospective cohort/17.7 years	NA (patients without DM or HF or AF or AMI)/24,349	62.50, 59.80	All-cause mortality	ICD-10	< 6.98 6.98–7.63 > 7.63	Ref HR: 0.96 (0.92–1.01) 1.17 (1.12–1.22)	Significant demographics, past comorbidities and medications
				AMI		< 6.98 6.98–7.63 > 7.63	Ref HR: 0.87 (0.79–1.06) 1.59 (1.45–1.74)	
				Cardiovascular death		< 6.98 6.98–7.63 > 7.63	Ref HR: 0.89 (0.79–1.03) 1.59 (1.41–1.79)	
Kim et al. [18]	Prospective cohort/5.7 years	Kangbuk Samsung Health Study dataset (participants without DM)/255,508	37.63, 47.37	All-cause mortality	ICD-10	Per 1 unit	HR: 0.97 (0.90–1.03)	Age, sex, BMI, SBP, LDL cholesterol, daily alcohol consumption, regular physical activity, current smoking, HOMA-IR
				Cardiovascular mortality		Per 1 unit	HR: 1.04 (0.88–1.25)	
(1)Liu et al. [19]	Prospective cohort/10.0 years	12 communities in Eastern China (community population without CVD or diabetes mellitus)/6095	48.69, 50.90	CVD (CHD and stroke)	ICD	Per 1 unit Per SD (NA) ≤ 7.92 7.92–8.32 8.32–8.76 > 8.76	HR: 1.24 (1.04–1.47) HR: 1.15 (1.03–1.28) Ref HR: 1.13 (0.81–1.57) 0.94 (0.67–1.32) 1.48 (1.07–2.05)	Age, gender, WHR, tobacco use, alcohol use, education, physical activity, hypertension, BMI, LDL-C, intake of fat and carbohydrates, use of antihypertensive drugs, and use of antilipemic drugs
				CAD		Per 1 unit Per SD (NA) ≤ 7.92 7.92–8.32 8.32–8.76 > 8.76	HR: 1.31 (1.05–1.63) HR: 1.19 (1.03–1.37) Ref HR: 1.23 (0.79–1.90) 1.00 (1.64–1.57) 1.69 (1.11–2.58)	
				stroke		Per 1 unit Per SD (NA) ≤ 7.92 7.92–8.32 8.32–8.76 > 8.76	1.20 (0.92–1.56) 1.12 (0.95–1.33) Ref HR: 1.21 (0.74–1.97) 1.03 (0.62–1.71) 1.40 (0.85–2.31)	
Quiroga et al. [20]	Prospective cohort/3.8 years	NEFRONA (persons without DM)/1142	59.00, 40.00	MACE (cardiovascular death, nonfatal MI, nonfatal stroke and hospitalization for unstable angina)	ICD	Per 1 unit < 8.63 ≥ 8.63	HR: 1.94 (1.01–3.73) Ref HR: 2.54 (1.27–5.07)	Age, sex, hypertension and atherosclerotic score
Zhao et al.[21]	Retrospective cohort/4.0 years	Beijing Anzhen Hospital (patients without diabetes who were diagnosed with NSTE-ACS and treated with elective PCI in 2015)/1510	59.70, 26.30	Cardiovascular events (all-cause death, nonfatal MI, nonfatal ischemic stroke, and ischemia-driven revascularization)	ICD	Per 1 unit < 8.72 ≥ 8.72	HR: 2.43 (1.85–3.20) Ref HR: 2.09 (1.60–2.72)	Age, gender, BMI, smoking history, hypertension, dyslipidemia, previous history of MI, PCI, stroke, PAD, diagnosis, TC, HDL-C, eGFR, HbA1c, LVEF, LM disease, three-vessel disease, chronic total occlusion, diffuse lesion, in-stent restenosis, SYNTAX score, treatment of LM, LCX, RCA, DES implantation, DCB application, complete revascularization, number of stents, DAPT at admission, statins at admission, and ACEI/ARB at discharge
				All-cause death		Per 1 unit < 8.72 ≥ 8.72	HR: 0.60 (0.12–3.11) Ref HR: 0.56 (0.13–2.50)	
				Nonfatal MI		Per 1 unit < 8.72 ≥ 8.72	HR: 3.54 (1.91–6.57) Ref HR: 1.97 (1.09–3.55)	
				Nonfatal ischemic stroke		Per 1 unit < 8.72 ≥ 8.72	HR: 1.86 (0.69–5.05) Ref HR: 1.31 (0.52–3.27)	
Yang et al.[22]	Prospective cohort/1.0 years	Abnormal Glucose Regulation in Patients with Acute Stroke across China (acute ischemic stroke patients without DM)/1226	62.00, 36.70	Stroke recurrence	ICD	4.31–5.48 5.48–5.81 5.81–6.22 6.22–8.17	Ref HR: 1.33 (0.80–2.23) 2.04 (1.26–3.31) 1.86 (1.13–3.06)	Age, sex, BMI, smoking status, medical history of hypertension, hyperlipidemia, AF and CAD, antihypertensive drugs, statins, intravenous alteplase, antiplatelet and anticoagulation during hospitalization, pulmonary infection and urinary infection during hospitalization, NIHSS at admission and TOAST subtypes
				All-cause mortality		4.31–5.48 5.48–5.81 5.81–6.22 6.22–8.17	Ref HR: 1.94 (1.09–3.48) 1.56 (0.83–2.93) 2.91 (1.62–5.23)	
Park et al. [23]	Prospective cohort study/4.2 years	Health risk assessment study and Korea Health Insurance Review and Assessment (Community population without DM)/16,455	46.10, 48.80	IHD (angina pectoris or acute MI)	ICD-10	≤ 8.08 8.09–8.45 8.45–8.85 ≥ 8.86	Ref HR: 1.61 (1.05–2.48) 1.85 (1.21–2.83) 2.28 (1.48–3.51)	Age, sex, BMI, smoking status, alcohol intake, physical activity, high sensitivity C-reactive protein, mean arterial blood pressure, C-reactive protein level, chronic kidney disease, and hypertension medication
Yang et al. [24]	Prospective cohort/2.4 years	Fuwai Hospital, Chinese National Center for Cardiovascular Diseases (nondiabetic patients who underwent PCI from January 2013 to December 2013)/5489	57.20, 20.60	MACE (all-cause death, nonfatal MI, nonfatal stroke, and target vessel revascularization)	ICD	Per 1 unit	HR: 0.79 (0.29–2.15)	Age, years, hyperlipemia, Previous stroke, previous MI, previous PCI, previous CABG, multivessel disease, CTO disease, SR disease, SYNTAX score, number of stents, LVEF, FBG, mmol/L, HbA1c, hs-CRP
Salazar et al. [27]	Prospective cohort/10.0 years	An epidemiological study was conducted in Rauch city, Buenos Aires, Argentina (RAUCH project, phase 2) (Community population without DM)/723	51.00, 67.20	Cardiovascular events (angina pectoris, MI, myocardial revascularization, and fatal or nonfatal stroke)	ICD	Per 1 unit < 8.98 ≥ 8.98	HR: 1.46 (1.03–2.08) Ref HR 1.93 (1.04–3.58)	Age, sex, smoking, LDL-C, BMI, and aspirin, antihypertensive and lipid-lowering drug use
Sun et al. [29]	Retrospective cohort/3.0 years	Beijing Anzhen Hospital (HF patients without DM undergoing elective PCI between June 2017 and June 2019)/1263	NA	MACE (all-cause mortality, non-fatal MI, and any revascularization)	ICD-10	< 8.54 8.54–8.93 8.93–9.41 ≥ 9.41	Ref HR: 1.30 (0.97–1.74) 1.70 (1.25–2.30) 1.71 (1.20–2.45)	Age, sex, heart rate, BMI, NYHA class, prior PCI, platelet, albumin, TC, LDL-C, HDL-C, potassium, uric acid, LVEF, ARB, thiazide diuretics, spironolactone, sacubitril/valsartan, diffuse lesion, SYNTAX score, LM disease, in-stent restenosis, target vessel, complete revascularization
Wu et al. [31]	Prospective cohort/1.0 year	Second Afliated Hospital of Guangzhou Medical University (nondiabetic patients with small vessel occlusion and acute ischemic stroke)/1970	66.46, 31.73	Ischemic stroke recurrence	ICD	< 8.20 8.20–8.53 8.54–8.92 > 8.92	Ref OR: 2.60 (1.28–5.25) 3.84 (1.85–7.95) 3.10 (1.37–8.02)	Age, lipid-lowering agents, fasting blood glucose, BMI, SBP, diastolic blood pressure, total cholesterol, triglyceride, HDL-C, LDL-C, uric acid and infarct locations, gender, smoking, drinking, hypertension, antihypertensive agents, antiplatelet agents, anticoagulant agents, stroke or transient ischemic attack history and CAD history
Wu et al. [30]	Retrospective cohort/5.8 years	Qilu Hospital of Shandong University, Shandong Provincial Hospital and The Second Hospital of Shandong University (nondiabetic patients after CABG)/830	62.79, 25.70	MACE (cardiac rehospitalization, ischemia-driven revascularization, non-fatal ischemic stroke, non-fatal MI, cardiac death, and all cause death)	ICD-10	Per 1 unit Per SD (NA) 7.78–8.45 8.45–8.84 8.84–9.50	HR: 1.85 (1.36–2.50) HR: 1.38 (1.18–1.62) Ref HR: 1.15 (0.73–1.81) 2.22 (1.46–3.38)	Age, sex, previous MI, previous stroke, previous PCI, left main disease, multivessel disease, BMI, LVEF, smoking, drinking, hypertension, hyperlipidemia, FH-CAD, duration of surgery, of-pump coronary artery bypass grafting, number of grafts, use of arterial grafts, eGFR, TC, LDL-C, HDL-C, European System for Cardiac Operative Risk Evaluation score II and medication use
				All-cause death		Per 1 unit Per SD (NA) 7.78–8.45 8.45–8.84 8.84–9.5	HR: 1.63 (0.95–2.79) HR: 1.29 (0.97–1.72) Ref HR: 1.55 (0.68–3.56) 2.92 (1.29–6.61)	
				Non- fatal MI		Per 1 unit Per SD (NA) 7.78–8.45 8.45–8.84 8.84–9.5	HR: 1.43 (0.77–2.67) HR: 1.21 (0.87–1.68) Ref HR: 0.87 (0.39–1.96) 1.30 (0.60–2.81)	
				Non-fatal stroke		Per 1 unit Per SD (NA) 7.78–8.45 8.45–8.84 8.84–9.5	2.16 (1.20–3.86) 1.50 (1.10–2.04) Ref HR: 0.87 (0.34–2.27) 2.36 (1.04–5.36)	
Yu et al. [26]	Prospective cohort/7.0 years	China Health and Retirement Longitudinal Study (participants without DM)/7760	58.00, 46.44	Stroke	ICD	Per 1 unit	HR: 1.14 (1.01–1.29)	Age, sex, education, smoking status, drinking status, SBP, diastolic blood pressure, BMI, low density lipoprotein cholesterol, high density lipoprotein cholesterol, hypertension, dyslipidemia, and hs-CRP

AMI: acute-myocardial infarction; BMI: body mass index; HT his: history of hypertension; PLT: platelet; UA: uric acid; ICD: International Classification of Diseases; ALP: alkaline phosphatase; LVEF, left ventricle ejection fraction; TC: total cholesterol; TyG: triglyceride and glucose index; LDL-C: low-density lipoprotein cholesterol; HDL-C: high-density lipoprotein cholesterol; ACEI: angiotensin-converting enzyme inhibitor; ARB: angiotensin receptor blocker; HF: heart failure; CAD: coronary artery disease; CKD: chronic kidney disease; IHD: ischemic heart disease; CHF: congestive HF; MACCE: major adverse cardio-cerebral events; IABP: intra-aortic balloon pump; SBP: systolic blood pressure; DBP: diastolic blood pressure; HbA1c: Glycated hemoglobin A1c; TG: triglyceride; FBG: fasting plasma glucose; ICH: intracranial hemorrhage; AF: atrial fibrillation; CHD: coronary heart disease; eGFR: estimated glomerular filtration rate; LM: left main coronary artery; CABG: coronary artery bypass graft; ARB: angiotensin receptor blocker; ACEI: angiotensin-converting enzyme inhibitor; NT-proBNP: N-terminal pro-B-type natriuretic peptide; PCI: percutaneous coronary intervention; hs-CRP: high sensitivity C-reactive protein; bSS: baseline SYNTAX score; rSS: residual SYNTAX score; ACS: acute coronary syndrome; STEMI: ST-elevation myocardial infarction; UA: unstable angina; SA: stable angina; Scr: serum creatinine; PVD: peripheral vascular disease; DAPT: dual antiplatelet therapy; SYNTAX: synergy between PCI with taxus and cardiac surgery; DCB: drug-coated balloon; T2DM: type 2 diabetes mellitus; PAD: peripheral artery disease; PTCA: percutaneous transluminal coronary angioplasty; CTO: chronic total occlusion; Cr: creatinine; WBC: white blood cell; DPP-4i: dipeptidyl peptidase-4 inhibitor; LAD: left anterior descending; PIR: poverty income ratio; WHR: waist hip ratio; NIHSS: National Institutes of Health Stroke Scale; TOAST: Trial of Org 10,172 in Acute Stroke Treatment; NSTE-ACS: non-ST-segment elevation acute coronary syndrome; OPCABG: of-pump coronary artery bypass grafting; EuroSCORE II: European System for Cardiac Operative Risk Evaluation score II; USA: United States of America; NA: not application

Only 3 studies had an NOS score of 6 [22, 46, 66], indicating potential issues with selection and outcome bias. The other studies had scores between 7 and 9, indicating acceptable quality (Online Table S5).

### Associations between the TyG index and CVD events and mortality

#### Cardiovascular events

Forty-three cohort studies (DM = 23; non-DM = 26) with 7,172,313 participants [6, 14, 16–32, 36–42, 44–46, 48–50, 56–61, 63–65, 67, 69] were included. The mean age of the participants was 31.45 years (DM: 62.98; non-DM: 31.23), and 40.66% of participants were female (DM: 35.78%; non-DM: 40.70%). After the studies conducted by Cho et al. and Kim et al. [16, 18] were excluded, the mean age of participants without diabetes increased to 57.57 years.

**Fig. 2:**
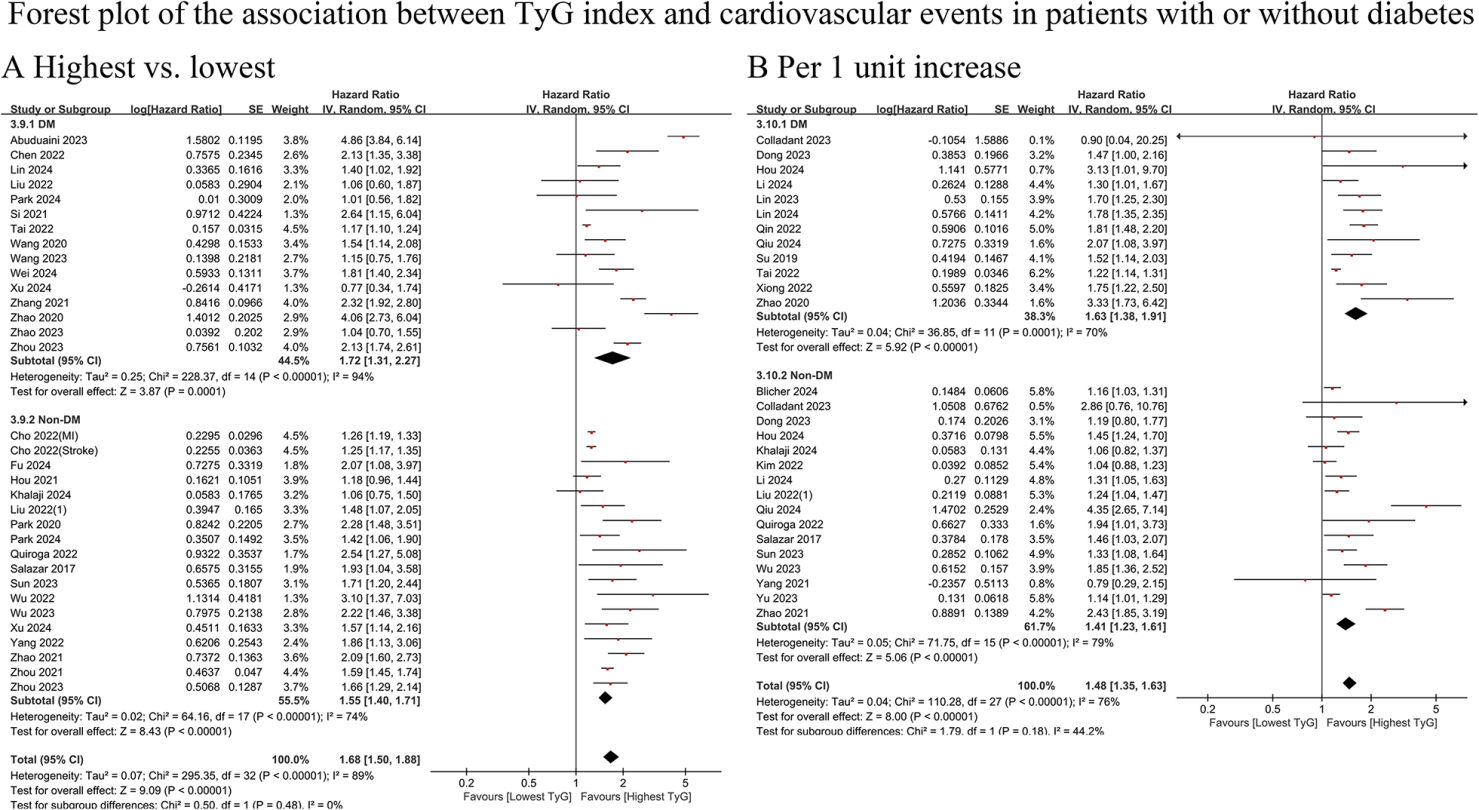
Forest plot of the association between TyG index and cardiovascular events in patients with or without diabetes. **A** cardiovascular events, analyzed as category variables (highest vs. lowest). **B**, cardiovascular events, analyzed as continuous variables (per 1 unit increase). TyG, triglyceride-glucose; DM, diabetes mellitus

The results indicated that TyG was significantly associated with a greater risk of cardiovascular events in patients with diabetes (highest vs. lowest HR: 1.72, 95% CI: 1.31–2.27, I^2^ = 94%, τ2 = 0.25, Q test *P* < 0.00001; median vs. lowest HR: 1.32, 95% CI: 1.15–1.52, I^2^= 75%, τ2 = 0.05, Q test *P* < 0.00001) or without diabetes (highest vs. lowest HR: 1.55, 95% CI: 1.40–1.71, I^2^ = 74%, τ2 = 0.02, Q test *P* < 0.00001; median vs. lowest HR: 1.18, 95% CI: 1.06–1.31, I^2^ = 77%, τ2 = 0.02, Q test *P* < 0.00001). The risk of CVD events associated with the TyG index was similar between those with diabetes and those without diabetes (highest vs. lowest: *P* = 0.48; median vs. lowest: *P* = 0.19) (Fig. 2A and Online Figure S1A). This result remained consistent when the TyG index was considered a continuous variable (HR for DM vs. non-DM: 1.63 vs. 1.41, *P* = 0.18) (Fig. 2B).

#### Composite of MACCEs

**Fig. 3:**
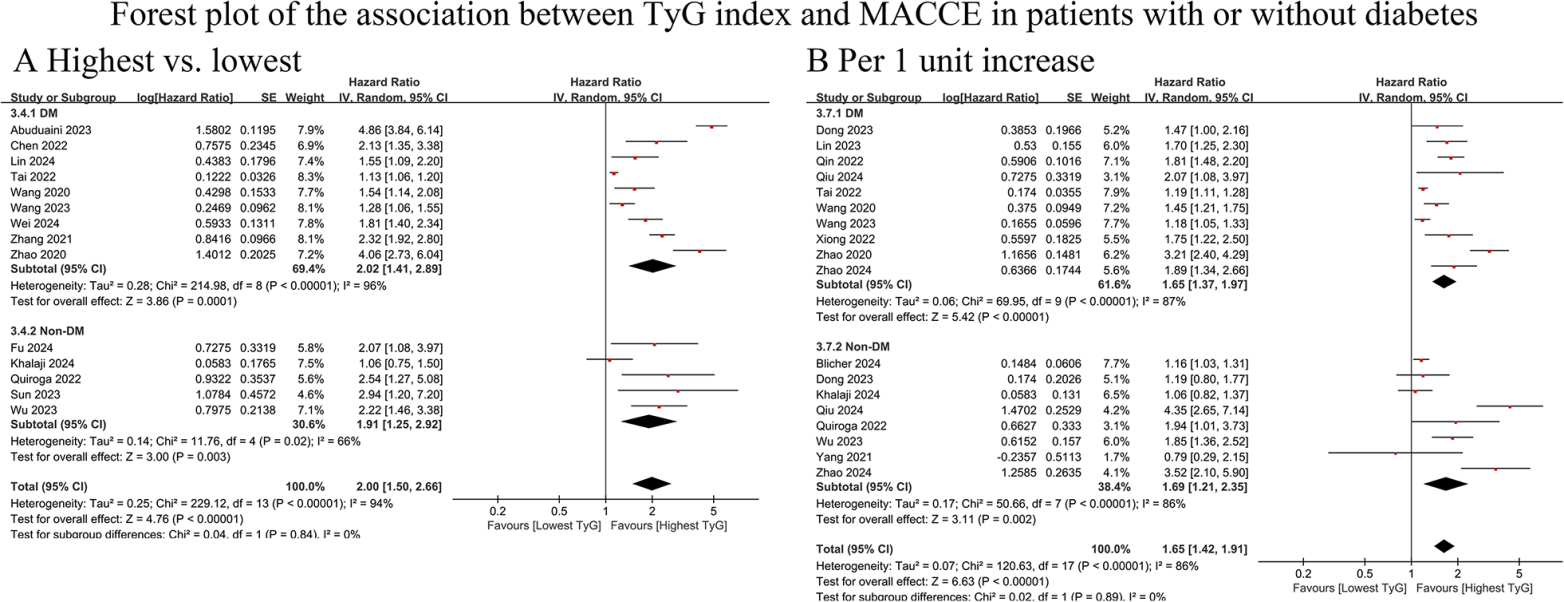
Forest plot of the association between TyG index and MACCE in patients with or without diabetes. **A** MACCE, analyzed as category variables (highest vs. lowest). **B** MACCE, analyzed as continuous variables (per 1 unit increase). TyG, triglyceride-glucose; DM, diabetes mellitus; MACCE, major adverse cardio-cerebral event

Twenty-two cohorts (DM = 15; non-DM = 10) were included, with 54,058 participants [14, 20, 24, 29, 30, 37, 39, 41, 42, 44, 46, 48–50, 58–61, 64, 65, 67, 69]. The findings indicated that TyG was significantly associated with a greater risk of MACCEs in both diabetic (highest vs. lowest HR: 2.02, 95% CI: 1.41–2.89, I^2^ = 96%, τ2 = 0.28, Q test *P* < 0.00001) and nondiabetic (highest vs. lowest HR: 1.91, 95% CI: 1.25–2.92, I^2^ = 66%, τ2 = 0.14, Q test *P* = 0.02) patients. The TyG index-associated risk of MACCEs was similar between diabetic and nondiabetic individuals (*P* = 0.53) (Fig. 3A). This result remained consistent when the TyG index was considered a continuous variable (HR for DM vs. non-DM: 1.65 vs. 1.69, *P* = 0.89) **(**Fig. 3B).

#### Stroke

**Fig. 4:**
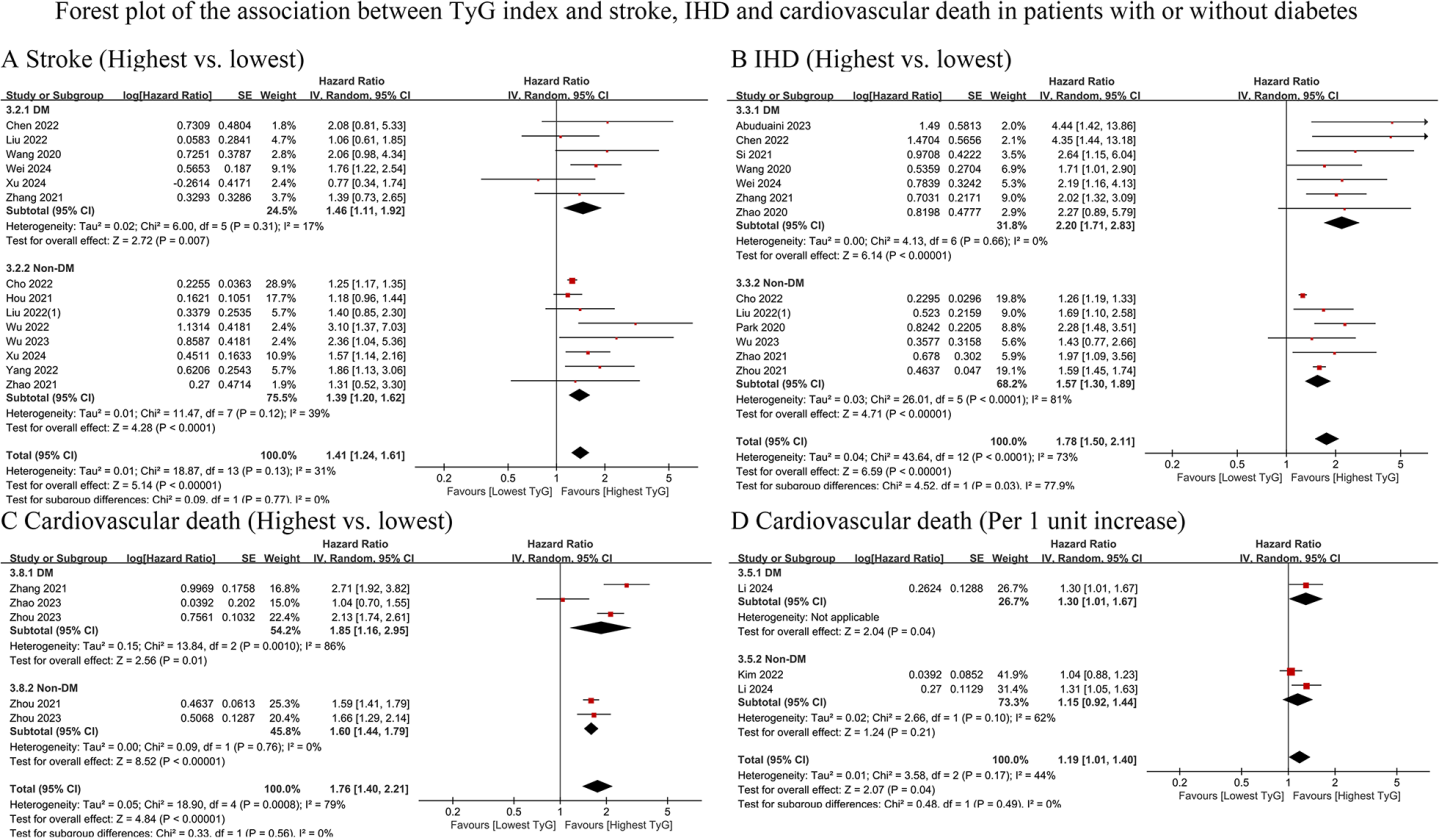
Forest plot of the association between TyG index and stroke, IHD and cardiovascular death in patients with or without diabetes. **A** stroke, analyzed as category variables (highest vs. lowest). **B** IHD, analyzed as category variables (highest vs. lowest). **C** cardiovascular death, analyzed as category variables (highest vs. lowest). **D** cardiovascular death, analyzed as continuous variables (per 1 unit increase). TyG, triglyceride-glucose; DM, diabetes mellitus; IHD, ischemic heart disease

Fourteen cohort studies (DM = 6; non-DM = 9) with 6,729,335 participants were included [14, 16, 17, 19, 21, 22, 26, 30, 31, 45, 48, 63, 64, 67]. The findings showed that TyG was significantly associated with a greater risk of stroke in both diabetic (highest vs. lowest HR: 1.46, 95% CI: 1.11–1.92, I^2^ = 17%, τ2 = 0.02, Q test *P* = 0.31) and nondiabetic (highest vs. lowest HR: 1.39, 95% CI: 1.20–1.62, I^2^ = 39%, τ2 = 0.01, Q test *P* = 0.12) patients. The TyG index-associated stroke risk was similar in patients with and without diabetes in the comparison of the highest and lowest TyG index groups (*P* = 0.77) **(**Fig. 4A**)**.

#### Ischemic heart disease

Thirteen cohort studies (DM = 7; non-DM = 6) with 6,734,656 participants were included [14, 16, 19, 21, 23, 28, 30, 48, 49, 57, 60, 64, 67]. The results indicated that TyG was significantly associated with a greater risk of IHD in both diabetic (highest vs. lowest HR: 2.20, 95% CI: 1.71–2.83, I^2^ = 0%, τ2 = 0, Q test *P* = 0.66) and nondiabetic (highest vs. lowest HR: 1.57, 95% CI: 1.30–1.89, I^2^ = 81%, τ2 = 0.03, Q test *P* < 0.0001) patients. The association between the TyG index and IHD risk was stronger in diabetic patients than in nondiabetic patients (*P* = 0.03) **(**Fig. 4B**)**.

#### Cardiovascular death

Six cohort studies (DM = 4; non-DM = 4) with 319,126 participants were included [14, 18, 28, 36, 38, 56]. The results revealed that TyG levels were significantly associated with a greater risk of cardiovascular death in both diabetic (highest vs. lowest HR: 1.85, 95% CI: 1.16–1.95, I^2^ = 86%, τ2 = 0.15, Q test *P* = 0.001) and nondiabetic (highest vs. lowest HR: 1.60, 95% CI: 1.44–1.79, I^2^ = 0%, τ2 = 0.00, Q test *P* = 0.76) patients. The TyG index-associated cardiovascular death risk estimate was similar in patients with or without diabetes, with the highest vs. lowest risk (*P* = 0.56) **(**Fig. 4C**)**. This result remained consistent when the TyG index was considered a continuous variable (HR for DM vs. non-DM: 1.30 vs. 1.15, *P* = 0.49) **(**Fig. 4D**)**.

#### All-cause mortality

**Fig. 5:**
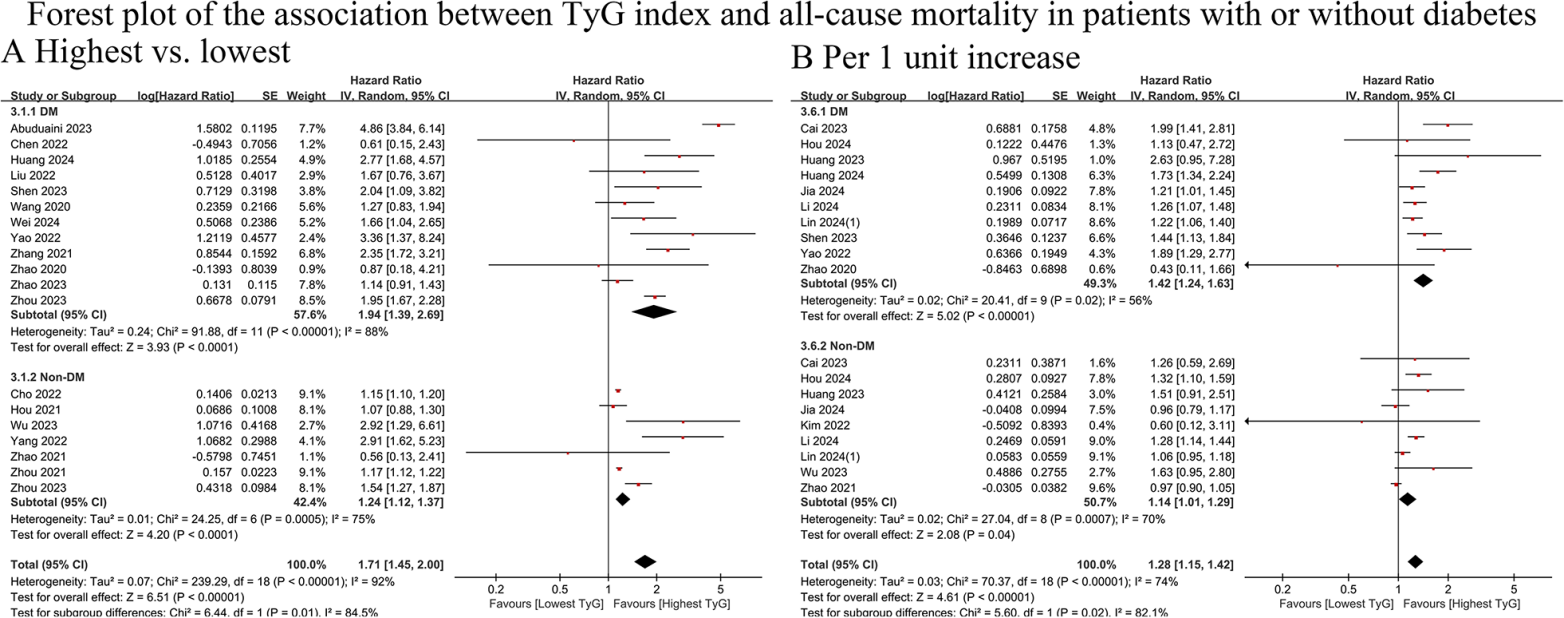
Forest plot of the association between TyG index and all-cause mortality in patients with or without diabetes. **A** all-cause mortality, analyzed as category variables (highest vs. lowest). **B** all-cause mortality, analyzed as continuous variables (per 1 unit increase). TyG, triglyceride-glucose; DM, diabetes mellitus

Twenty-five cohort studies (DM = 17; non-DM = 14) with 7,137,948 participants were included [14, 16–18, 21, 22, 28, 30, 33–36, 38, 40, 43, 48, 49, 56, 60, 62–64, 66–68]. The pooled results revealed that TyG was significantly associated with a greater risk of all-cause mortality in both diabetic (highest vs. lowest HR: 1.94, 95% CI: 1.39–2.69, I^2^ = 88%, τ2 = 0.24, Q test *P* < 0.00001; median vs. lowest HR: 1.45, 95% CI: 1.17–1.81, I^2^ = 73%, τ2 = 0.07, Q test *P* = 0.0001) and nondiabetic (highest vs. lowest HR: 1.24, 95% CI: 1.12–1.37, I^2^ = 75%, τ2 = 0.01, Q test *P* = 0.0005; median vs. lowest HR: 1.03, 95% CI: 0.97–1.10, I^2^ = 55%, τ2 = 0.00, Q test *P* = 0.04) patients. Moreover, the association between the TyG index and all-cause mortality was stronger in diabetic patients than in nondiabetic patients (highest vs. lowest: *P* = 0.01; median vs. lowest: *P* = 0.003) (Fig. 5A and Online Figure S1B). This result remained consistent when the TyG index was considered a continuous variable (HR for DM vs. non-DM: 1.42 vs. 1.14, *P* = 0.02) (Fig. 5B).

#### Assessment of quality and publication bias

Funnel plots and Egger’s test indicated potential publication bias for cardiovascular events in nondiabetic patients, whereas no such bias was detected in diabetic patients. Egger’s test revealed publication bias for cardiovascular events in the nondiabetic group (*P* = 0.002), but it did not affect the overall findings after the trim-and-fill analysis (Online Figure S2).

Due to the high heterogeneity in cardiovascular events, MACCEs, cardiovascular death, IHD, and all-cause mortality, their ratings were downgraded. The GRADE assessment revealed a low level of certainty for outcomes related to MACCEs, cardiovascular death, and all-cause mortality in patients with and without DM, as well as for cardiovascular events and IHD in non-DM patients. It indicated a moderate level of certainty for cardiovascular events and IHD in DM patients and for stroke in patients with and without DM (Online Table S6).

#### Sensitivity analysis and subgroup analyses

Furthermore, sensitivity analysis by omission of each individual study demonstrated the stability of the association between the TyG index and cardiovascular events in participants with DM. After omitting the study with the largest sample size by Cho et al. [16], the heterogeneity decreased from I² = 74% (Q test for *P* < 0.00001) to I² = 49% (Q test for *P* = 0.01).

Subgroup analyses revealed the cardiovascular event associations were stronger in groups with smaller sample sizes (HR: 1.18 vs. 1.90, *P* = 0.001), shorter follow-up times (HR: 1.27 vs. 1.97, *P* = 0.03), and retrospective studies (HR: 1.24 vs. 2.62, *P* < 0.0001) (Online Table S7) of participants with DM. The association between the TyG index and cardiovascular events did not significantly differ by mean age or diabetes severity in participants with DM. Consistently, the association was stronger in groups with smaller sample sizes (HR: 1.39 vs. 1.82, *P* = 0.009), and a borderline significant association was detected in retrospective studies (HR: 1.40 vs. 1.68, *P* = 0.06) in participants without DM (Online Table S7). The associations did not significantly differ by mean age, follow-up duration, or study design among participants without DM.

## Discussion

### Major findings

By pooling study-level data from 50 cohorts involving 7,239,790 participants, we showed that the TyG index was associated with an increased risk of cardiovascular events and mortality independent of diabetes. Moreover, the risk estimates for IHD and all-cause mortality among diabetic patients were significantly greater than those among nondiabetic patients. Taken together, these results indicate that diabetes is a significant modifier of the associations between the TyG index and IHD as well as all-cause death. To our knowledge, this study is the first to comprehensively investigate the diabetes-specific associations of the TyG index with cardiovascular events and mortality.

### Comparison with previous studies

TyG index, which is calculated from routine fasting triglyceride and glucose levels, has been widely recognized for its associations with cardiovascular outcomes across diverse populations. Nevertheless, most available evidence, including findings from this study, has relied on baseline values. Research investigating the utility of monitoring changes in the TyG index over time remains limited, although some emerging studies have highlighted its potential value. A prospective cohort study of 20,185 elderly participants, followed for a mean of 4.25 years, demonstrated that elevated baseline TyG index levels and trajectories of medium stability or gradual increase were associated with increased CVD risk [70]. Additionally, a retrospective cohort study of 233,546 participants demonstrated that increasing TyG index levels during follow-up were independently associated with increased risks of all-cause and cardiovascular mortality [71].

The assessment of MACCEs traditionally relies on multiple biomarkers, including high-sensitivity cardiac troponin T, lipid profiles, high-sensitivity C-reactive protein, and N-terminal pro-B-type natriuretic peptide (NT-proBNP). Compared with specialized cardiac biomarkers, such as high-sensitivity cardiac troponin T and NT-proBNP, the TyG index is cost effective and has increased accessibility, particularly in resource-limited settings. Compared with acute cardiac biomarkers, the TyG index has an additional role in cardiovascular and metabolic health. A prospective cohort with 15.4 years of follow-up demonstrated the incremental predictive value of the TyG index when integrated into the traditional CVD risk score in 1,970 adults without CVD, diabetes, or antihypertensive or lipid-lowering treatment [41]. A 10-year prospective cohort study demonstrated that integrating changes in the TyG index into the Framingham risk model enhanced the ability to predict incident CVD among 5,014 participants without preexisting CVD [72]. Among individuals with diabetes or established CVD, the incremental predictive value of the TyG index remains contentious. A prospective cohort study of 1,932 consecutive T2DM patients with acute myocardial infarction who were followed for a median of 26.8 months demonstrated that incorporating the TyG index into a baseline risk model significantly enhanced the predictive value for MACCEs [14]. A 16-year prospective cohort study of 7,521 Iranian participants with a high prevalence of T2DM and hypertension revealed that, for individuals under 60 years of age, integrating the TyG index into the Framingham risk score did not significantly enhance predictive ability [73]. Future studies are needed to assess the prognostic value of the TyG index when incorporated into the conventional risk score in individuals with CVD or diabetes.

The relationships between the TyG index and the incidence of cardiovascular events and mortality have been studied within the general population. However, few studies have specifically addressed diabetes-related associations with cardiovascular diseases. A cross-sectional study involving 1,516 subjects revealed that the TyG index-associated risk of carotid atherosclerotic plaques was similar in patients, regardless of whether they had diabetes (OR for DM vs. non-DM: 1.52 vs. 2.81) [74]. A retrospective cohort study revealed no significant difference in MACCE rates among patients with acute coronary syndrome who had a history of coronary artery bypass grafting and subsequently underwent percutaneous coronary intervention (*P* = 0.57) [46]. Furthermore, a meta-analysis of 4 studies revealed that diabetes status did not significantly influence the relationship between the TyG index and the occurrence of atherosclerotic cardiovascular diseases (*P* = 0.79) [8]. Our findings demonstrated a stronger association between the TyG index and IHD in diabetic patients than in nondiabetic patients, somewhat in contrast to previous findings. Nonetheless, because diabetes is a recognized risk factor for coronary arteriosclerotic heart disease, this outcome is not unexpected. As an alternative measure for IR, which is prevalent in patients with T2DM, these findings were anticipated. T2DM status may amplify the association between the TyG index and IHD. Our study revealed that cardiovascular events and mortality were not significantly different between diabetic and nondiabetic patients. Since the definition of cardiovascular events in the present study included heart failure and/or cardiovascular death, a potential reason may be that TyG is more strongly associated with the incidence of IHD in diabetic patients but not with the prognosis of patients with established CVD. However, this assumption should be confirmed by prospective studies.

Significant heterogeneity was observed in all the outcomes. Several important confounders should be considered, particularly age. Specifically, patients with diabetes tend to be older than those without diabetes. The average age of the participants was 63.90 years for the DM group and 31.23 years for the non-DM group. The higher risk estimates for cardiovascular events and mortality among patients with diabetes may be attributed to this age difference, although almost all studies had adjusted for age. To account for the influence of age, we excluded two studies with the largest nondiabetic populations [16, 18], resulting in a significant reduction in the age difference (65.18 for DM, 57.57 for non-DM). This sensitivity analysis demonstrated consistent results. In patients with DM, higher risk estimates were found for IHD (*P* = 0.03) and all-cause mortality (*P* = 0.01). Another factor was that patients with diabetes tend to have worse health conditions and comorbidities than those without diabetes do, and these factors may not have been adequately adjusted for, which may have led to a stronger association between the TyG index and the risk of cardiovascular events and mortality.

Sensitivity analysis revealed that the Cho et al. [16] study, the largest cohort included in this study with 6,675,424 patients, potentially influenced the heterogeneity of the association between the TyG index and cardiovascular events in participants without DM, suggesting that sample size variations may contribute to the observed heterogeneity. The results of subgroup analyses indicate that the association was stronger in groups with smaller sample sizes and retrospective studies, which may contribute to the heterogeneity of the results in both diabetic and nondiabetic patients. The majority of the smaller sample studies included in our analysis focused on patients with CVD or those undergoing cardiovascular surgery. Retrospective studies may be susceptible to selection and survival biases, systematically inflating risk assessments by overrepresenting severe cases and relying on medical records that more comprehensively document significant CVD events or adverse outcomes. This study design and sample size selection, where complex and extreme medical histories are more likely to be meticulously documented, may generate artificially stronger associations. These findings necessitate large-scale prospective cohort studies involving well-matched patients with and without diabetes to validate our findings.

### Clinical implications and perspective

As a simple and easily measurable indicator, the TyG index is likely to be more frequently utilized in clinical practice. This study revealed that the TyG index is independently linked to cardiovascular events and mortality in patients without diabetes. Moreover, a significant disparity in IHD and all-cause mortality was observed between patients with and without diabetes. These findings highlight the need for further research to elucidate the role of diabetes in the relationship between the TyG index and CVD incidence. Conversely, the present results also indicate that the TyG index may act as a stronger independent predictor of increased IHD risk in patients with diabetes.

Notable challenges should be addressed before the TyG index can be confidently used as a routine monitoring tool. One significant limitation is the lack of large-scale, prospective studies explicitly designed to evaluate the predictive value of TyG index trajectories for MACCEs. In addition, longitudinal changes in the TyG index may be influenced by a range of confounding factors, including lifestyle modifications, pharmacological interventions, and comorbid conditions, which may limit the interpretability of the results. Furthermore, the lack of standardized cutoffs or thresholds for interpreting changes over time diminishes its clinical applicability. These gaps in the literature highlight the need for further investigation. Future research should focus on developing standardized protocols for monitoring and interpreting these changes, which is critical for integrating the TyG index into existing cardiovascular risk assessment frameworks. Studies should also explore the impact of therapeutic interventions, such as surgical interventions, lifestyle modifications, and pharmacological treatments, on TyG trajectories and examine whether such changes are associated with clinically meaningful reductions in MACCE risk.

### What do we update?

Previous studies and our reports revealed that the TyG index is associated with a greater risk of cardiovascular diseases, atherosclerotic cardiovascular diseases, and subclinical cardiovascular diseases [7–11]. Nevertheless, no studies have reported the impact of diabetes status on the relationship between the TyG index and CVD risk. Compared with previous meta-analyses, the present study revealed that a higher TyG index is independently associated with a greater risk of IHD and all-cause mortality in individuals with DM than in those without DM. Collectively, these results suggest that diabetes significantly modified TyG-associated IHD and all-cause mortality.

### Strengths and limitations

This meta-analysis is the first to explore the impact of diabetes on the association between the TyG index and cardiovascular events. Moreover, this study was based on cohort studies, a design that may help minimize recall bias. Nonetheless, the present study has certain limitations. First, as a meta-analysis of cohort studies, this study does not allow for the establishment of causality. Despite the inclusion of studies that employed multivariate analysis, fully controlling for residual confounding variables, such as age, comorbidities, follow-up duration, and medications, is not feasible. Second, outcomes differed among the 50 studies included, leading to the limited number of studies available for each outcome, which included individuals with and without diabetes. The applicability of these findings to other regions and ethnic groups remains uncertain. Finally, the study populations differ significantly, with some including individuals with cardiovascular disease and others drawn from the general population, resulting in considerable differences in cardiovascular event rates. The considerable heterogeneity in baseline characteristics and therapeutic regimens necessitates validation across diverse populations.

## Conclusion

In conclusion, the TyG index was associated with a higher risk of cardiovascular events and mortality in patients with or without diabetes. A stronger association between the TyG index and IHD and all-cause mortality was observed in diabetic patients than in those without diabetes. Diabetes is a significant modifier of the association between IHD and all-cause death. Nevertheless, the limited number of studies underscores the necessity for prospective studies to explore this association further in diverse populations. This study suggests that future studies should consider the role of diabetes in the TyG index-associated CVD events and mortality.

## Electronic supplementary material

Below is the link to the electronic supplementary material.

Supplementary Material 1

## Data Availability

No datasets were generated or analysed during the current study.

## Abbreviations

TyG: Triglyceride–glucose
DM: Diabetes mellitus
MACCE: Major adverse cardiovascular and cerebrovascular events
HR: Hazard ratio
IHD: Ischemic heart disease
T2DM: Type 2 diabetes mellitus
IR: Insulin resistance
HIEG: Hyperinsulinemic–euglycemic
CVD: Cardiovascular disease
MACE: Major adverse cardiovascular events
PRISMA: Preferred reporting items for systematic reviews and meta-analyses
CAD: Coronary artery disease
GRADE: Recommendations assessment, development, and evaluation
NOS: Newcastle–Ottawa scale
CIs: confidence intervals
τ2: Tau^2^
NT-proBNP: N-terminal pro-B-type natriuretic peptide
