# Supplementary figures and images for: Does diabetes modify the triglyceride–glucose index associated with cardiovascular events and mortality? A meta-analysis of 50 cohorts involving 7,239,790 participants
Source: Cardiovasc Diabetol. 2025 Jan 27;24:42. doi: 10.1186/s12933-025-02585-z (PMC11773825; doi:10.1186/s12933-025-02585-z)

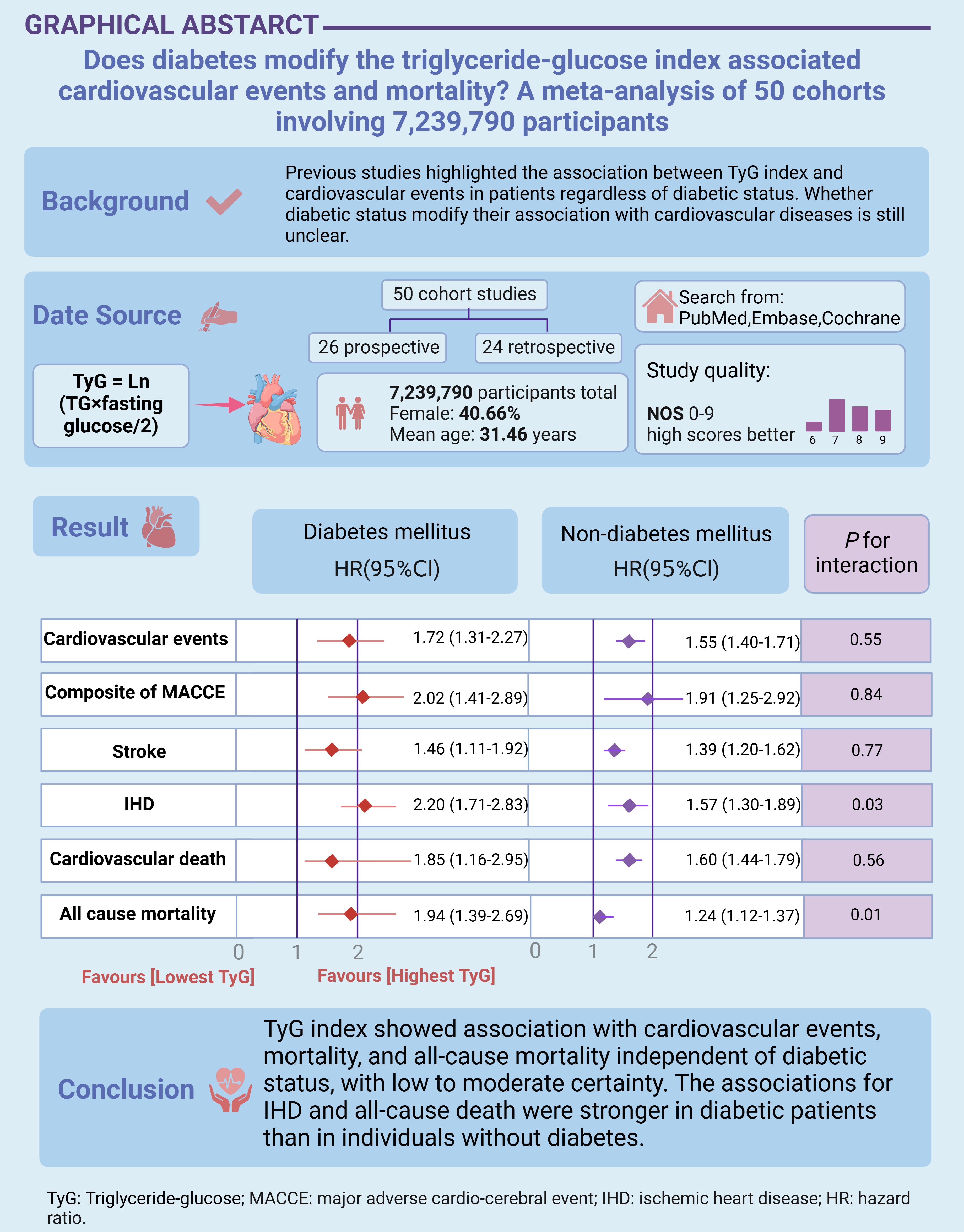

Supplement: Supplementary file 1 — Supplementary Material 1 [file 12933_2025_2585_MOESM1_ESM.png]
